# Opinions of the Press

**Published:** 1887-12-10

**Authors:** 


### THE NATIONAL PENSION FUND.

### OPINIONS OF THE PRESS.

**Freeman's Journal.**

The lines of a mutual assurance society are closely followed, the fundamental rule being kept in mind of making the contributions of the members sufficient to defray the sick allowances and pensions, as calculated by well-known actuaries. Only thus could financial stability be secured. The scheme certainly claims the sympathy and support of all who appreciate the noble work done by those whose mission is to relieve suffering, as well as those who desire to see habits of thrift promoted amongst the people. We are anxious to know what part, if any, Ireland will occupy in regard to the scheme. If once taken up by a few influential members of the medical profession, there should be no difficulty in bringing the scheme under the notice of all in this country who possess the qualifications necessary for membership.

